# Multi-spectroscopic monitoring of molecular interactions between an amino acid-functionalized ionic liquid and potential anti-Alzheimer's drugs†

**DOI:** 10.1039/d0ra06323a

**Published:** 2020-10-23

**Authors:** Srishti Sharma, Manoj Kumar Banjare, Namrata Singh, Jan Korábečný, Kamil Kuča, Kallol K. Ghosh

**Affiliations:** School of Studies in Chemistry, Pt. Ravishankar Shukla University Raipur-492010 C.G. India kallolkghosh@gmail.com; MATS School of Sciences, MATS University Pagaria Complex, Pandri Raipur-492009 C.G. India; Ramrao Adik Institute of Technology, DY Patil University Nerul Navi Mumbai India; Biomedical Research Center, University Hospital Hradec Kralove Sokolska 581 500 05 Hradec Kralove Czech Republic kamil.kuca@uhk.cz; Department of Toxicology and Military Pharmacy, Faculty of Military Health Sciences, University of Defence Trebesska 1575 500 01 Hradec Kralove Czech Republic; Department of Chemistry, Faculty of Science, University of Hradec Kralove Rokitanskeho 62 50003 Hradec Kralove Czech Republic

## Abstract

Inhibiting the formation of amyloid fibrils is a crucial step in the prevention of the human neurological disorder, Alzheimer's disease (AD). Ionic liquid (IL) mediated interactions are an expedient approach that exhibits inhibition effects on amyloid fibrils. In view of the beneficial role of ILs, in this work we have explored complexation of anti-Alzheimer's drugs (*i.e.*, tacrine and PC-37) and an amino acid-functionalized IL [AIL (4-PyC8)]. Maintaining standard physiological conditions, the binding mechanism, thermo-dynamical properties and binding parameters were studied by employing UV-vis, fluorescence, FTIR, ^1^H NMR, COSY and NOESY spectroscopy. The present investigation uncovers the fact that the interaction of anti-Alzheimer's drugs with 4-PyC8 is mediated through H-bonding and van der Waals forces. The Benesi–Hildebrand relation was used to evaluate the binding affinity and PC-37 showed the highest binding when complexed with 4-PyC8. FTIR spectra showed absorption bands at 3527.98 cm^−1^ and 3527.09 cm^−1^ for the PC-37 + 4-PyC8 system which is quite promising compared to tacrine. ^1^H-NMR experiments recorded deshielding for tacrine at relatively higher concentrations than PC-37. COSY investigations suggest that anti-Alzheimer's drugs after complexation with 4-PyC8 show a 1 : 1 ratio. The cross-peaks of the NOESY spectra involve correlations between anti-Alzheimer's drugs and AIL protons, indicating complexation between them. The observed results indicate that these complexes are expected to have a possible therapeutic role in reducing/inhibiting amyloid fibrils when incorporated into drug formulations.

## Abstract 2

Ionic liquids mediated interactions are an expedient approach that exhibit inhibition effect on amyloid fibrils which is beneficial for the treatment of Alzheimer's disease.

## Graphical abstract

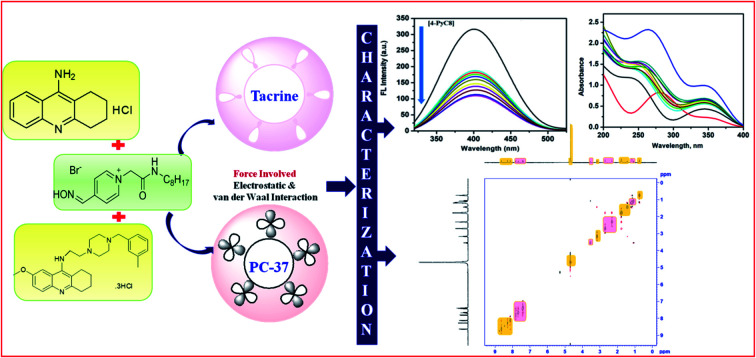

## 1. Introduction

In the last few decades, targeted drug delivery has become a crucial issue in the bio-medical field as it provides the undeniable advantage of minimizing the associated after-effects of drugs like psychotic illness, overstimulation and other dysfunctions.^1,2^ Generally, drugs interact with surfactants, ionic liquids (ILs), cyclodextrins and other carriers to promote their controlled and specific delivery.^3–5^ Earlier ILs were recognized only as better alternatives to volatile organic solvents with superior properties.^6^ However, the better biodegradability and non-toxic profile of functionalized ILs have attracted biochemists, ecologists and medical scientists worldwide. Recent studies conducted on ILs with incorporated functional groups have proved their biological efficacy, inhibiting or enhancing enzyme activities.^7^ Garcia *et al.* have studied such biodegradable and amino acid-functionalized ILs (AILs), which possess a superior surface activity and a very low critical micelle concentration (CMC).^8^ Functionalization of ILs with amide groups leads to the elevation of their thermal stability and enhancement of their self-aggregation properties due to the elongation of the alkyl chain and escalated antimicrobial activity. Recently, our research group has reported the synthesis of a series of unique ILs derived from an amphiphilic pyridinium oxime moiety, which were examined on the grounds of biodegradability using a closed bottle test.^9^ In addition to the aforementioned interesting properties, reports also evidence ILs' remarkable inhibitory effects for amyloid fibrils (nearly 50%).^10,11^ It has been shown in the literature that amino acids can inhibit/arrest the polymerization of amyloid fibrils, which are responsible for causing neurological disorders.^12,13^ Zarić *et al.* has critically reviewed the role of various aromatic amino acids and their approach towards the inhibition of amyloid formation.^14^ Hence, a proper understanding of the interaction between drugs and AILs can be used to modify the drug's solubilization and enable them to cross the blood–brain barrier (BBB). Furthermore, it could prove beneficial to produce exciting results for prevailing neurological disorders which lack potent drugs.^15,16^

Alzheimer's disease (AD) is a neurological disorder which significantly imbalances the socio-economy of the world.^17^ AD is broadly defined as memory loss, consequently leading to a continuous decline in thinking capacity that disrupts a person's ability to function independently.^18^ Despite a long history of intensive research, AD still lacks efficient medical counter measures and potent anti-Alzheimer's drugs.^19^ However, a few anti-Alzheimer drugs have been approved by the US Food and Drug Administration (FDA), but those are helpful only for short-term symptomatic relief and are accompanied by various side-effects.^20,21^ Tacrine (1,2,3,4-tetrahydroacridin-9-amine) was the first drug approved by the FDA in 1993; it is a reversible AChE inhibitor that interacts with the α-anionic site of the enzyme. However, it was discontinued in 2013 due to its hepatotoxicity.^22^ Despite this, the tricyclic structure of tacrine is still preferred for the design of new anti-Alzheimer's drug candidates serving as a template inhibitor.^23,24^ Likewise, PC-37 (7-methoxy-*N*-(2-{4-[(3-methylphenyl)methyl]piperazin-1-yl}ethyl)-1,2,3,4-tetrahydroacridin-9-amine trihydrochloride) is a cholinesterase inhibitor conjugating 7-methoxytacrine and donepezil templates into one chemical entity.^25^ The bioavailability expressed as AUC_total_ was 28 179 ± 4691 min ng mL^−1^ for PC-37 and it was found to be capable of targeting the central nervous system at elevated concentrations. Moreover, it is pertinent to mention here that the *in vivo* experiments showed no evidence of toxicity reports, which further corroborates the potential utility of PC-37 for AD treatment.^26^ Therefore, it is expected that the complexes formed between the AIL and anti-Alzheimer's drugs could be of benefit and will be revolutionary in the development of modified anti-Alzheimer's drugs.

Considering the imminent features of AILs and the requirement of potent anti-Alzheimer's drugs, herein, we have investigated the binding interaction of anti-Alzheimer's drugs, namely tacrine (the standard inhibitor) and PC-37 (a synthesized anti-Alzheimer's drug) with AIL *i.e.*, 4-((hydroxyimino)methyl)-1-(2-(octylamino)-2-oxoethyl)pyridin-1-ium bromide (4-PyC8) at 310 K and 7.5 pH. By employing UV-vis, steady-state fluorescence, FTIR, ^1^H NMR, COSY and NOESY spectroscopy, the inclusion complexation, characterization and binding ability of this interaction has been studied both in aqueous and solid-state. Although several investigations on drug–IL interactions are available, as per the authors' knowledge, the complexation between anti-Alzheimer's drugs and AILs is rather limited. To meet this point, this is the very first attempt of such a study. This complexation is a novel approach to modify drugs *via* functionalized ILs and make them more efficient as drug formulations for AD treatment. Mostly, this study is expected to provide valuable insights and more in-depth knowledge about the interaction of AIL with anti-Alzheimer's drugs. In the future, these complexes could help to inhibit the formation of amyloid fibrils in AD.

## 2. Experimental section

### 2.1 Source and purity of the samples

**Scheme 1:**
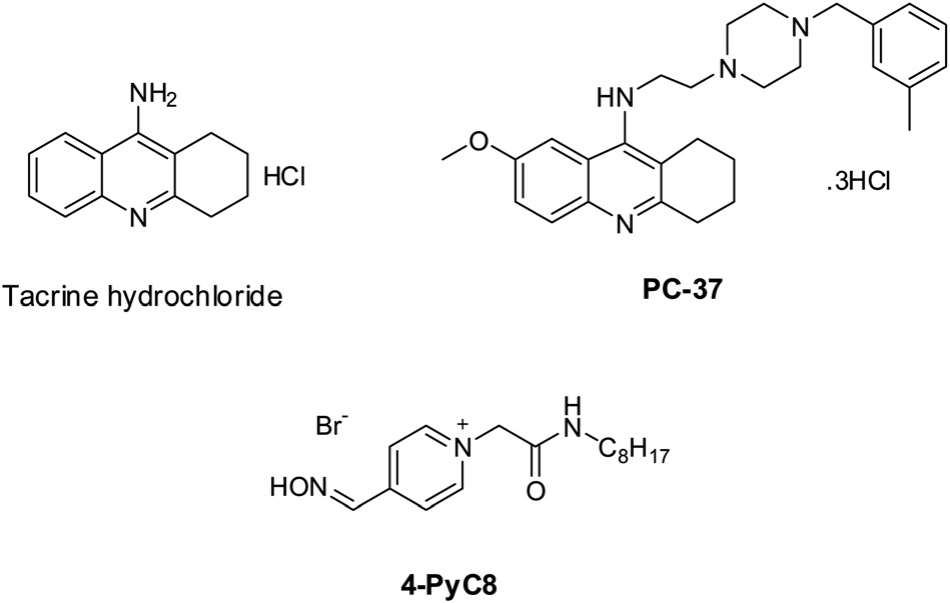
The chemical structures of the potential anti-Alzheimer's drugs (tacrine and PC-37) and the amino acid-functionalized ionic liquid (4-PyC8).

Tacrine is commercially available (Sigma-Aldrich, Prague, Czech Republic). PC-37 was synthesized at the Biomedical Research Center, University Hospital Hradec Kralove, Czech Republic. The synthesis of PC-37 was in line with the formerly reported protocol.^24^ 4-PyC8 was obtained from Dr Yevgen Karpichev (Department of Chemistry and Biotechnology, Tallinn University of Technology (TalTech), Tallinn-12618, Estonia). The chemical structures of the anti-Alzheimer's drugs and AIL are represented in Scheme 1. Tris buffer GR [tris(hydroxymethyl) aminomethane] and sodium hydroxide (NaOH) (≥97.0%) were purchased from Merck, Mumbai, India. Deuterium oxide (D_2_O) was purchased from Merck KGaA, Darmstadt, Germany. Potassium bromide (KBr) (99.0%) was purchased from Sigma-Aldrich, Bangalore, India. The chemical reagents were used in the same condition as purchased without further purification. All the solutions were freshly prepared with double distilled water and were incubated for 15 min before being put into the instrument.

### 2.2 Preparation of the anti-Alzheimer's drugs-AIL complex

The complexes were prepared using the co-precipitation method.^27^ 0.1 g of the anti-Alzheimer's drugs (tacrine and PC-37) was dissolved in 10 mL double distilled water. AIL (0.1 mM) in 10 mL water was slowly added to the drug solution with continuous stirring. The molar ratio of AIL to anti-Alzheimer's drugs was 1 : 1. The vessel was covered with aluminum foil and stirred continuously for 48 h at 305 K before being refrigerating at 278 K. The precipitated compound was recovered by filtration and washed with a small amount of water to remove unfused particles. Double distilled water was used to prepare all the solutions. pH measurements were recorded using a pH meter (pH 700 EUTECH Instruments).

## 3. Methods

### 3.1 UV-vis spectroscopy

The UV-vis absorption spectra of the potential anti-Alzheimer's drugs-AIL complexes were recorded using a Varian Cary-60 (Agilent Technology) UV-vis spectrophotometer. Scans were taken in the wavelength range of 200–800 nm in a 1.0 cm path length quartz cuvette after 15 minutes from the addition of the AIL at 310 K.

### 3.2 Fluorescence spectroscopy

Steady state fluorescence measurements for anti-Alzheimer's drugs-AIL complexes were performed with a Cary Eclipse fluorescence spectrophotometer (Agilent Technology). An excitation wavelength of 300 nm was used and the emission spectra were scanned from 310 nm to 600 nm. Both excitation and emission band slits were fixed at 5 nm. All data were acquired using a quartz microcuvette of 10 mm.

### 3.3 Fourier transform infrared spectroscopy

FTIR spectrum was recorded on a Nicolet iS10 spectrometer (Thermo Fisher Scientific Instrument, Madison, USA) by using a KBr matrix. All the spectra were taken *via* the diffuse reflectance Fourier transform infrared (DRS-FTIR) method with a resolution of 4 cm^−1^ and 32 scans at room temperature in the mid IR range of 400–4000 cm^−1^.

### 3.4 Proton nuclear magnetic resonance spectroscopy

^1^H NMR spectra were recorded using a Bruker NMR spectrometer operating at 400 MHz using saline D_2_O (protonated signal at 4.79 ppm) as an internal standard. The chemical shift values for these complexes were determined and the signals were quoted as *δ* values in ppm.

## 4. Results and discussion

### 4.1 Binding of the anti-Alzheimer's drugs with AIL

The binding between any two compounds is fundamentally confirmed and monitored by the UV-vis technique.^28^ Absorption spectra of such an interaction are demonstrated to investigate the extent of the interaction between anti-Alzheimer's drugs (tacrine and PC-37) and AIL (4-PyC8) in aqueous solutions. The absorption spectrum measurements are recorded in the range of 200–600 nm. All the molecular interactions experiments were performed under physiological conditions (310 K and 7.5 pH) to ensure their relevance as drug delivery tools in the near future. Potential anti-Alzheimer's drugs (0.1 mM) gave characteristic peaks at *λ*_max_ = 400 and 375 nm wavelengths for tacrine and PC-37, respectively. In the case of the absorption spectra of 4-PyC8 (0.1 mM), the wavelength of maximum absorbance appears at *λ*_max_ = 400 nm. On the addition of AIL the absorption intensities of the drugs increase however, after reaching a maximum concentration no further augmentation was seen in the spectral intensities. It is known that after entering into a hydrophobic environment from an aqueous media, drugs usually show a red shift in their absorption maxima. The absorption spectra of tacrine and PC-37 in aqueous solutions with increasing concentrations of 4-PyC8 are shown in Fig. S1.† The increasing spectral intensities indicate the interaction between the anti-Alzheimer's drugs (tacrine and PC-37) and AIL (4-PyC8) and also the formation of a new complex (tacrine + 4-PyC8 and PC-37 + 4-PyC8). These interactions were substantially manifested by H-bonding, electrostatic interaction hydrophobic and van der Waals forces. Apart from an enhancement of the absorption intensities, no other shifting or deviations were observed either in the case of tacrine or PC-37.

Determination of the interaction parameters is necessary for such complexes, hence, they are evaluated using the Benesi–Hildebrand eqn (1).^29^1
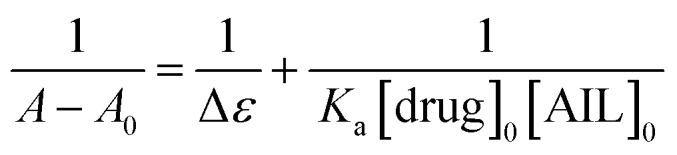
where *A* is the absorbance of the anti-Alzheimer's drug in the presence of AIL and *A*_0_ is the absorbance of the anti-Alzheimer's drug in the absence of AIL. Δ*ε* is the difference in the molar absorptivity of the anti-Alzheimer's drug in the presence and absence of the AIL.^30^*K*_a_ is the association constant while [drug]_0_ and [AIL]_0_ represent the respective concentrations of the drug and AIL.

The 1/(*A* − *A*_0_) *versus* 1/[AIL] plot gives a straight line as shown in Fig. S1† for the anti-Alzheimer's drug (tacrine and PC-37) and AIL (4-PyC8). The association constant *i.e.*, *K*_a_ was calculated from the ratio of the intercept and the slope of the Benesi–Hildebrand plot. The association constant values are evaluated to claim the stability of the complexation as they are the parameters that signify the formation of a complex in solution. The association constant is used to affirm the strength of the interaction between the molecules that interact together to form the complex. The values of the association constants are given in Table 1. The PC-37 + 4-PyC8 shows a greater value of *K*_a_ which evidences that the PC-37 + 4-PyC8 complex has a stronger binding affinity than tacrine + 4-PyC8. This could plausibly be due to the more hydrophobic character of PC-37 compared to tacrine (clog *P*_PC-37_ = 4.88 *vs.* clog *P*_tacrine_ = 2.63; calculated using Marvin Sketch software 17.17.0), which makes them responsible for the competing binding efficiencies. The molecular structure of tacrine comprises a tricyclic core, whereas PC-37 possesses an additional benzyl appendage with a 3-methyl moiety which contributes to its hydrophobicity. These facts support the better binding profile of PC-37 over tacrine.

**Table 1:** The association constant (*K*_a_) and Gibb's free energy (Δ*G*) for the binding of tacrine and PC-37 with 4-PyC8 in aqueous medium

Drug + AIL	*K*_a_ (M^−1^)	Δ*G* (kJ mol^−1^)
Tacrine + 4-PyC8	45.45 × 10^5^	−37.98
PC-37 + 4-PyC8	41.49 × 10^6^	−43.46

### 4.2 Thermodynamics of binding

The thermodynamics of complexation were further taken into consideration as they hold significant information.^31^ As quantitative evidence is crucial to evaluate in complexation studies by calculating the exact amount of energy changes involved in the process, some relevant information can be obtained regarding the feasibility of the binding event. An association constant is significantly related to the Gibb's free energy.^32,33^ Hence, Gibb's free energy changes, Δ*G*, for complexation can be obtained for such complexation using *K*_a_ according to eqn (2).

2 Δ*G* = −2.303*RT* log *K*_a_

where *R* is the gas constant (8.314 J mol^−1^ K^−1^), *T* is temperature in K and *K*_a_ is the association constant.

The negative value of Gibb's free energy assures that the binding is feasible. The association constant and the Gibb's free energy, Δ*G*, of the anti-Alzheimer's drugs-AIL complexes are summarized in Table 1.

### 4.3 AIL induced quenching of the intrinsic fluorescence of anti-Alzheimer's drugs

**Fig. 1:**
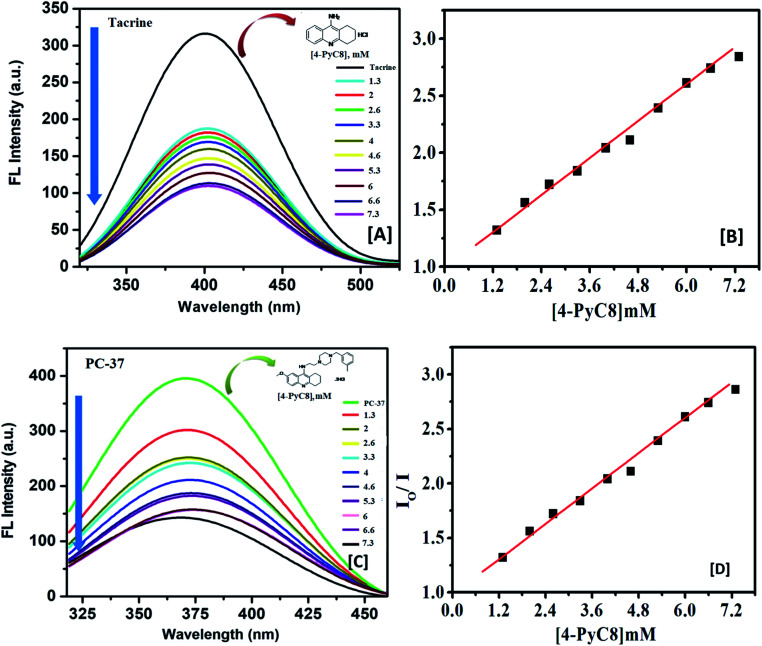
Fluorescence emission spectra (A and C) and Stern–Volmer plots (B and D) for the quenching of the fluorescence of the anti-Alzheimer's drugs (A and B) tacrine and (C and D) PC-37 with gradual addition of 4-PyC8 are displayed, respectively. The spectra were recorded at pH 7.5 and temperature 310 K (*λ*_ex_ = 300 nm and *λ*_ex_ = 310 nm, each slit width was adjusted to 5 nm).

Fluorescence techniques are extensively used for the studies of static and dynamic properties of different fluorescent systems either occurring by collision or due to the complex formation between the quencher and the fluorophore.^34^ Linear Stern–Volmer plots are known to represent a single quenching mechanism.^35^ This technique helps in calculating the binding constants and the number of binding sites which further explains the interaction between host and guest molecules.^36^ The fluorescence quenching method has been extensively studied for the determination of the binding parameters.^37^ Here, fluorescence measurements were performed to determine the thermodynamics of the interaction of anti-Alzheimer's drugs with AIL. The representative fluorescence emission spectra of anti-Alzheimer's drugs-AIL complexes are shown in Fig. 1. This was done to understand the effect of the functionalized IL on the recently developed anti-Alzheimer's drug environment and to evaluate the extent of the binding of their interaction. The intrinsic fluorescence of the drugs was measured in the presence of an increasing concentration of AIL while the concentration of anti-Alzheimer's drugs (0.1 mM) was kept constant in the solution.

Their interaction subsequently resulted in significant fluorescence quenching which indicates that anti-Alzheimer's drugs (tacrine and PC-37) bind to AIL (4-PyC8) in close vicinity. In both cases (tacrine and PC-37), the emission maximum was quenched, indicating the binding of the drugs to 4-PyC8 by altering their local environment with more pronounced quenching in the case of PC-37 (Fig. 1). Tacrine and PC-37 gave an emission peak at 400 and 375 nm, respectively. On the addition of 4-PyC8 in a concentration-based manner, the peak height is quenched simultaneously which eventually suggests the new complex formation between the respective drug and IL. In Fig. 1, the first spectrum is of the pure drug, tacrine (∼intensity 310 nm) at a zero concentration of AIL. Soon after the addition of AIL (2 μM), substantial quenching was observed at ∼170 nm. Likewise, for the spectra of PC-37 where the highest intensity spectrum is of the pure drug at zero concentration of AIL and the rest of the spectra quenches due to an increasing concentration of AIL. Both the systems show values of binding capacity more than unity, where the PC-37 + 4-PyC8 again shows a greater binding capacity over tacrine + 4-PyC8 (PC-37 + 4-PyC8 > tacrine + 4-PyC8). The magnitude of the binding constant implies that PC-37 + 4-PyC8 was quenched more possibly due to the presence of bulky substituent groups on their chemical structure, whereas tacrine serves less hindrance on their molecular structure.

The strength of the interaction between the quencher and fluoroprobe can be estimated using the Stern–Volmer quenching constant (*K*_SV_) also known as the first-order quenching rate constant. The *K*_SV_ is calculated from eqn (3), using the slope value illustrated in Table 2. The fluorescence quenching data is measured by using the classical Stern–Volmer relation.^38^

3 ln(*I*_0_/*I*) = 1 + *K*_SV_[Q]

Here, *I*_0_ and *I* represent the fluorescence intensities in the absence and presence of quencher Q, respectively. *K*_SV_ is the Stern–Volmer quenching constant and [Q] is the concentration of the quencher.

**Table 2:** The values of the Stern–Volmer quenching constants (*K*_SV_), association constants (*K*_a_) and regression constants (*R*^2^) for the anti-Alzheimer's drugs-AIL complex in aqueous medium

Parameters	Anti-Alzheimer's drugs + AIL	
	Tacrine + 4-PyC8	PC-37 + 4-PyC8
*K*_SV_ (10^4^ L mol^−1^)	11.17	23.35
*K*_a_ (10^4^ L mol^−1^)	13.05	79.52
*R* ^2^	0.998	0.999

The values of the number of binding sites (*n*) and the association constant *K*_a_ can be calculated from the fluorescence using the following eqn (4).4
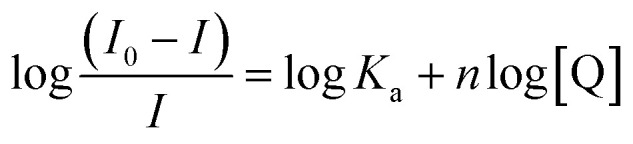
Here, *K*_a_ is the binding constant and *n* is the average number of binding sites. A plot of 
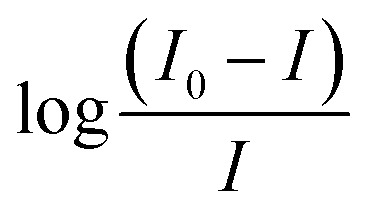
 against log[Q] is provided in Fig. S2.† The binding sites for both the complexes were found to be unity while the other values of binding constant and regression constant are tabulated in Table 2.

Table 2 indicates the relatively stronger interaction of PC-37 with 4-PyC8 at physiological temperature. It is observed that the slope increased upon the addition of an increasing concentration of AIL in 0.1 mM of the anti-Alzheimer's drugs, which indicates that the system is more susceptible to the quencher at high concentrations of AIL. As depicted in Fig. 1, PC-37 caused significant quenching after addition of 4-PyC8 which is in good agreement with the moderate binding constant (Table 2). On the contrary, tacrine showed relatively weaker binding profiles and therefore, was found to have a lower magnitude of binding constant. It can be inferred from the experimental observations that the PC-37 + 4-PyC8 system has significantly more binding followed by tacrine + 4-PyC8. Table 2 clearly shows that the *K*_SV_ value of the PC-37 + 4-PyC8 complex is greater than the tacrine + 4-PyC8 complex. Both the fluorescence and UV results support these observations of the complex formation and binding affinities.

### 4.4 Fourier transform infrared investigation

FTIR spectroscopy is a powerful analytical technique broadly classified as a useful tool for determining functional groups in the fields of food, environment, agriculture and especially pharmaceuticals.^39,40^ Using this technique, chosen drug samples can be analyzed with the aims of identification and localization of chemical type by FTIR in the transmission.^41^

#### (a) FTIR spectra of AIL

**Fig. 2:**
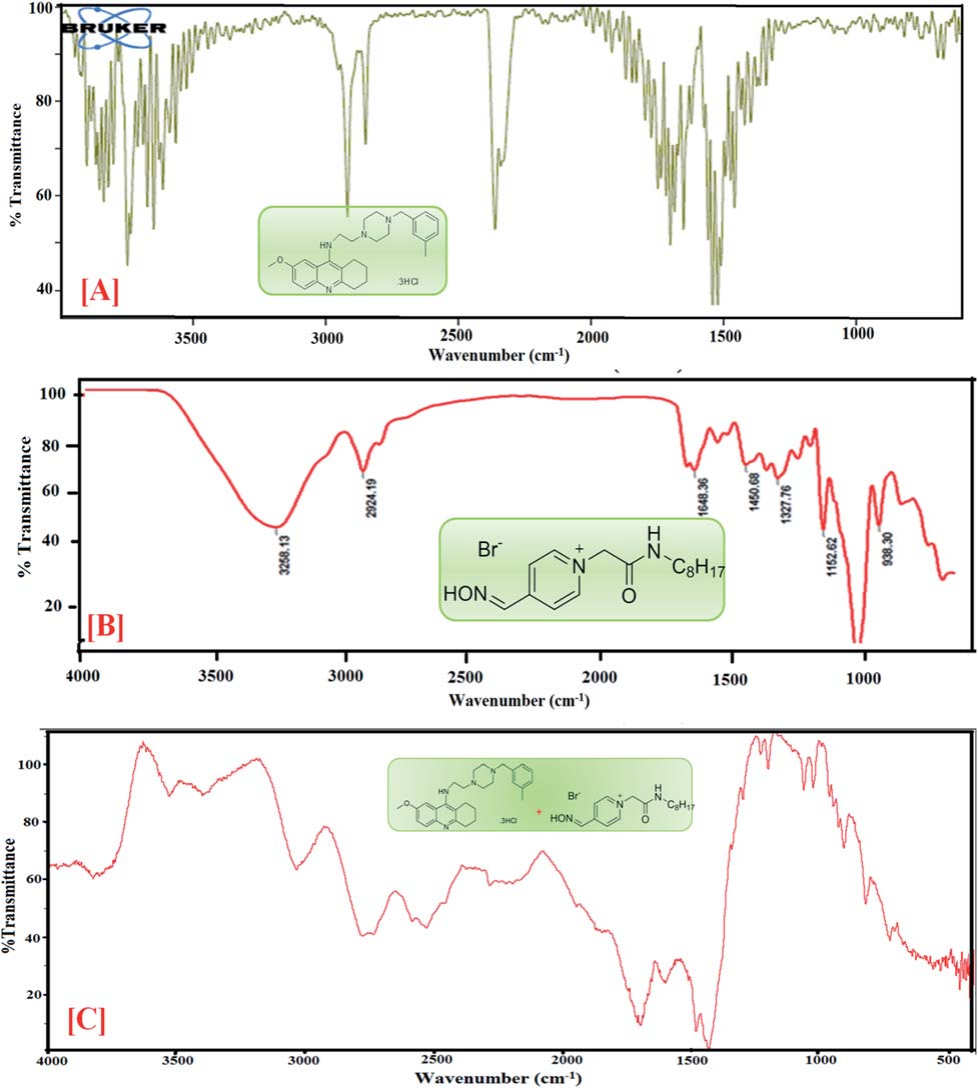
FTIR spectra of [A] PC-37, [B] 4-PyC8 and [C] PC-37 + 4-PyC8.

**Fig. 3:**
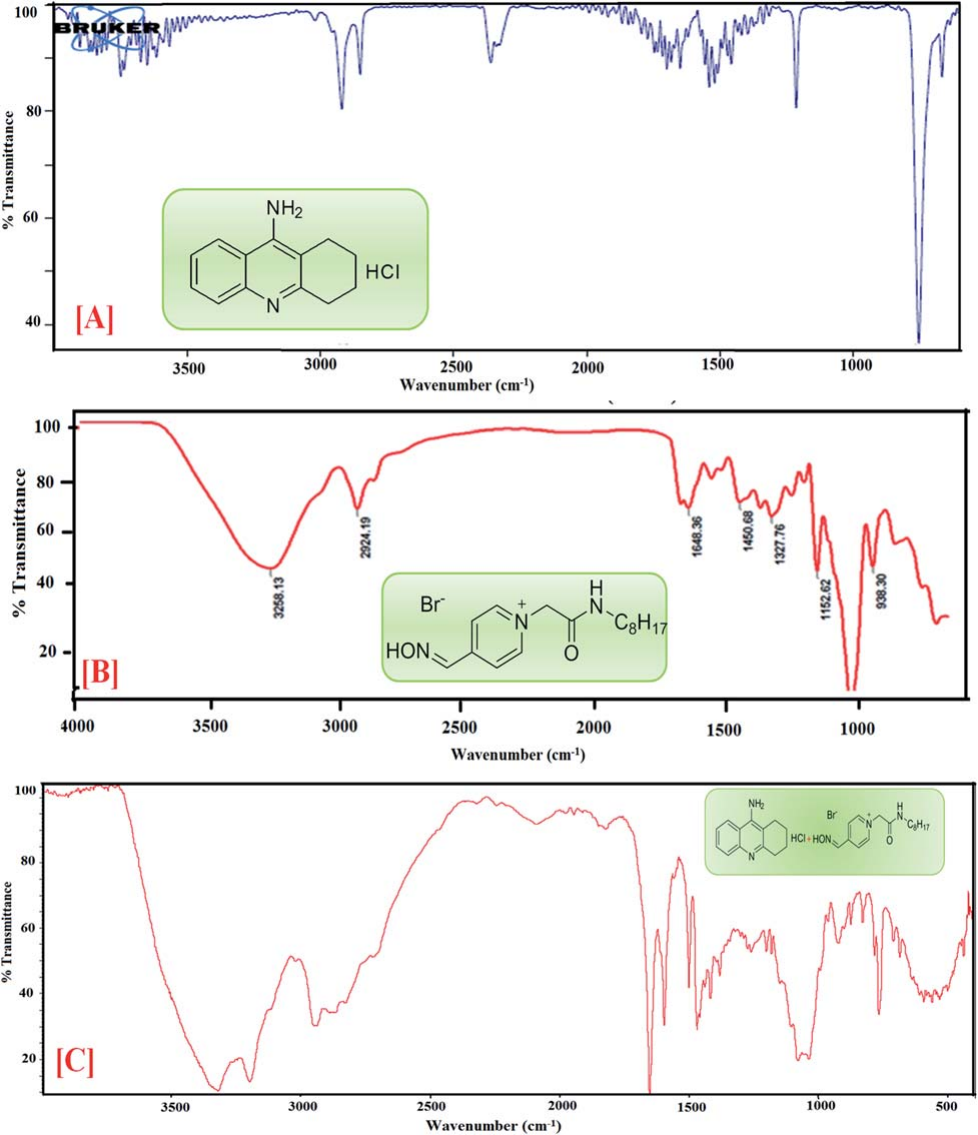
FTIR spectra of [A] tacrine, [B] 4-PyC8 and [C] tacrine + 4-PyC8.

According to the spectra of AIL (Fig. 2B and 3B), the absorption bands observed at 3400 to 3300 cm^−1^ correspond to the –NH stretching of amine, the absorbance peaks at 2848.72 and 2920 cm^−1^ correspond to the C–H stretching of the –CH_2_ group, the stretching frequency at 1658.66 cm^−1^ is attributed to the C=N of the oxime group, the absorption peaks at 1414 cm^−1^ show –OH bending, 1205 cm^−1^ indicates C=O stretching and absorption peaks at 832 cm^−1^ correspond to C–H bending. The featured IR stretching frequency of AIL is predicted in Table 3.

**Table 3:** Functional groups assigned to the interacting species of 4-PyC8 and tacrine + 4-PyC8 and PC-37 + 4-PyC8, respectively

Assignment groups	Wavenumber (cm^−1^)		
	4-PyC8	Tacrine	PC-37
N–H/O–H stretching	3299.09	3530.15	3565.78
Alkenyl C–H stretching	2920.30	2918.84	2917.80
C–OH stretching	2848.72	2480.15	2849.57
Aromatic C=C bending	1658.66	1540.61	1746.83
C–H bending and rocking	1414.08	1420.15	1419.29
C–O stretching	1205.99	1214.82	1396.97
=C–H bending	832.48	755.15	

#### (b) FTIR spectra of the anti-Alzheimer's drugs

The IR spectra of tacrine and PC-37 (Fig. 2A and 3A) show peaks at 3299.09 cm^−1^ and 3530.15 cm^−1^ corresponding to N–H stretching for amine and O–H stretching, respectively. (ii) Absorption peaks for C–H stretching are observed at 2920.30 cm^−1^ and 2918.84 cm^−1^, (iii) –OH stretching is found at 2848.72 cm^−1^ and 2480.15 cm^−1^, (iv) aromatic C=C bending is found at 1658.66 cm^−1^ and 1540.61 cm^−1^, (v) C–H bending and rocking peaks are at 1414.08 cm^−1^ and 1420.15 cm^−1^, (vi) 1205.99 cm^−1^ and 1214.82 cm^−1^ correspond to C–O stretching, and (vii) 832.48 cm^−1^ and 755.15 cm^−1^ are attributed to =C–H bending. The characteristic IR stretching frequencies of the anti-Alzheimer's drugs are predicted in Table 3.

#### (c) Interaction of anti-Alzheimer's drugs with AIL

The mixture of anti-Alzheimer's drugs and AIL show bands in the field ranging from 700 to 4000 cm^−1^. The absorption peaks of –NH stretching shifted to 3527 cm^−1^, C–H stretching at 3034 cm^−1^, –OH stretching at 2516 and 2530 cm^−1^, C=C bending at 1635 and 1703 cm^−1^, C–H stretching at 1423 and 1428 cm^−1^, C=O stretching at 1081 cm^−1^ and C–H bending at 823 cm^−1^ are shifted in the anti-Alzheimer's drugs-AIL complex. The FTIR data supports the successful complexation *via* absorbance of the excipients and the drugs. FTIR spectra of the pure PC-37, tacrine, AIL and their respective complexes are shown in Fig. 2 and 3. A comparative study was done to obtain information about the modification of the mixture spectra, meanwhile the proportion of AIL was increased. The characteristic IR stretching frequency of anti-Alzheimer's drugs with ILs is predicted in Table 4.

**Table 4:** Functional groups assigned for the interacting species of tacrine + 4-PyC8 and PC-37 + 4-PyC8, respectively

Assignment groups	Wavenumber (cm^−1^)	
	Tacrine + 4-PyC8	PC-37 + 4-PyC8
N–H/O–H stretching	3527.98	
Alkenyl C–H stretching	3034.48	3045.15
C–OH stretching	2516.97	2530.30
Aromatic C=C bending	1695.35	1703.35
C–H stretching bending and rocking	1428.59	1423.26
C–O stretching	—	1081.81
=C–H bending	823.05	

### 4.5 ^1^H NMR studies

^1^H NMR investigation for any binary system consisting of different moieties is capable of providing more in-depth information.^42^ Considering the accuracy of NMR, even a small shift of ∼0.01 ppm or higher is indicated as a significant change.^43^ Here, NMR chemical shifts were recorded for the two anti-Alzheimer's drugs *i.e.*, PC-37 and tacrine, in the absence and presence of AIL. The ^1^H NMR spectra clearly depict the interactions between the anti-Alzheimer's drugs (PC-37 and tacrine) and AIL (4-PyC8). The NMR titration experiments were performed using 0.01 mM of the anti-Alzheimer's drug solutions (PC-37 and tacrine), where AIL *i.e.*, 4-PyC8 was added in a concentration-based manner. All the solutions used in NMR experiment were prepared in D_2_O solvent. The alterations observed in the chemical shift (*δ*) values of the chosen anti-Alzheimer's drugs with the AIL solutions reveal the interactive nature between them.

After the addition of 1 mM of 4-PyC8, the changes in the *δ* values in the proton NMR spectra of the aliphatic and aromatic hydrogens of both the anti-Alzheimer's drugs along with the alterations in the aliphatic protons of 4-PyC8 are shown in Fig. S3.† At this concentration (1 mM) of AIL molecules the aromatic and aliphatic protons show upfield shifts. Increasing the AIL concentration up to 3 mM, the hydrogens of PC-37 and tacrine shift downfield implying that the surface is polar on the interface. After this, anti-Alzheimer's drugs come out at the surface of the complex as shown in Fig. S3.† Here, the anti-Alzheimer's drugs experience a deshielding effect through the positively charged pyridinium group, thus, the *δ* value is pushed to a higher magnitude. This is also evidenced from the UV-vis measurements. Tacrine experiences deshielding at higher concentrations (*i.e.* 1 mM) in comparison to PC-37 which consequently supports the earlier results obtained from UV-vis spectroscopy and fluorimetry, that the binding is higher in the case of the PC-37 + 4-PyC8 system followed by tacrine + 4-PyC8. The additional peaks found in the spectra may be due to the increased concentration of AIL.

The ^1^H NMR spectrum of the binary mixture of PC-37 + 4-PyC8 and tacrine + 4-PyC8 is presented in Fig. S3.† Two –CH_3_ groups were observed at ∼1.56 ppm and ∼2.32 ppm. Moreover, the aromatic protons' peaks were shown in the 7.8 ppm region as singlets. The ^1^H NMR spectra of the 4-PyC8 display some characteristics peaks. ‘*δ*’ values between 8–9 ppm indicate that the protons of the pyridinium ion are highly deshielded. Chemical shift values between 1–1.5 indicate the alkyl protons, which are highly shielded. Further, ‘*δ*’ values between 3.2–5.5 indicate the –NH and –CH=NH protons which are also deshielded.

The presence of 4-PyC8 brought significant alterations which are shown in terms of the *δ* values of α-CH_2_–, β-CH_2_– and –(CH_2_)_9_– sequential intensities of AIL. The prominent downfield shifting could be due to the deshielding effect of AIL. The α-CH_2_– shows a shift of ∼0.02 ppm. The –CH_2_– and –(CH_2_)_9_– sequential intensities of 4-PyC8 depict a significant shift of ∼0.08 ppm for the former and ∼0.07 ppm for the latter (Fig. S3†). Meanwhile, –CH_3_– was displaced by a ∼0.08 ppm shift. These reports together supported the existence of intermolecular interaction between the anti-Alzheimer's drugs-AIL complex.

### 4.6 COSY analysis

**Fig. 4:**
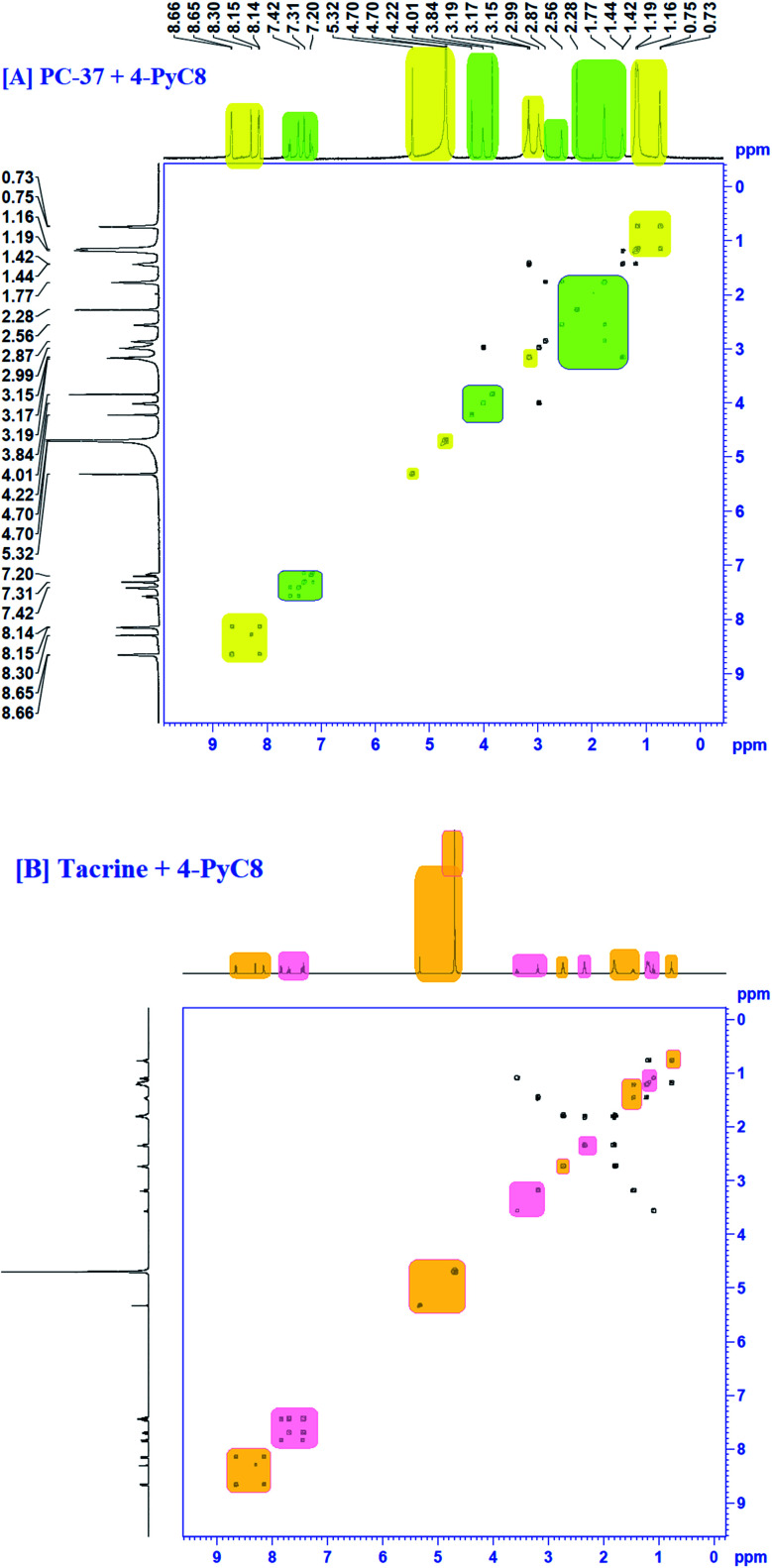
COSY spectra of complexation [A] PC-37 + 4-PyC8 and [B] tacrine + 4-PyC8.

COSY spectra were also used to confirm complexation. Fig. 4 explained the 2D COSY spectra of pure anti-Alzheimer's drugs and the complexes of anti-Alzheimer's drugs + AIL. It was found that there were no correlations recorded among the hydrogens of the –CH_3_ groups of 4-PyC8 and the protons of the anti-Alzheimer's drugs, signifying that the aromatic ring of 4-PyC8 forms the complex. Moreover, the proton signals of ^1^H NMR apparently change upon addition of anti-Alzheimer's drugs consequently, which referred to the interaction among the pyridine ring of the anti-Alzheimer's drugs and AIL. Although, the 2D COSY spectra show interactions between the oxime group of AIL and with the aromatic groups in the hydrocarbon chain of the anti-Alzheimer's drug molecules, which shows resonation in the center of 8–6 ppm, signifying that alteration was caused in the hydrocarbon chain of the AIL after it came under interaction with the anti-Alzheimer's drugs. As a result, we can conclude that the PC-37 protons interact more in comparison to tacrine with 4-PyC8. Additionally, by observing the COSY spectra we can find that there are correlations between the methylene groups of the AIL molecule resonating at 0.73–2.7 ppm. Here, long alkyl chains of the AIL were included and observed closer to the cavity of anti-Alzheimer's drugs. ^1^H–^1^H COSY spectra clearly show that only the –CH_3_ groups of 4-PyC8 were located in the anti-Alzheimer's drugs. Due to the overlapping with the alkyl chain of the AIL signal the cyclohexyl of PC-37 cannot be clearly distinguished. Conversely, after addition of the AIL molecule to the anti-Alzheimer's drugs solution, the aromatic ring becomes obvious and shifts upfield. Our COSY data are in agreement with the ^1^H-NMR and FTIR results. Based on this, the anti-Alzheimer's drugs with AIL are speculated to show complexation in a 1 : 1 ratio (Fig. 4). This present data on COSY analysis is new and interesting in the context of drugs–IL interaction studies.

### 4.7 NOESY analysis

**Fig. 5:**
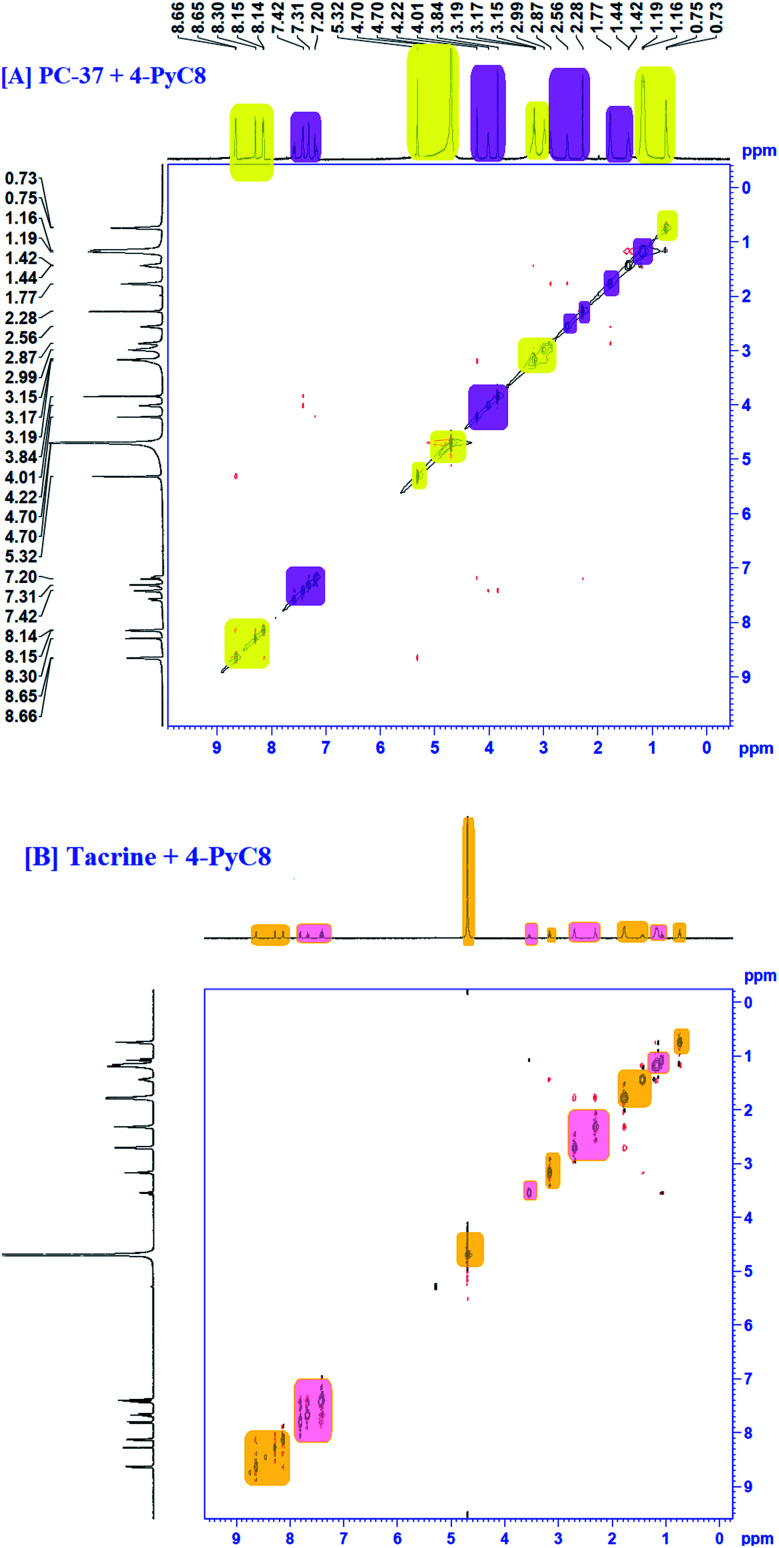
NOESY spectra of the complex of [A] PC-37 + 4-PyC8 and [B] tacrine + 4-PyC8.

To gather more information about the complexation, NOESY analysis was performed to investigate the closely spaced but non-bonded protons involved in the interaction. The substitution of AIL with D_2_O could be examined while comparing it with COSY data observed on a Bruker NMR instrument with respect to data recorded using a recent sample prepared in deuterium (Fig. 5). In the case of PC-37 + 4-PyC8/tacrine + 4-PyC8 in deuterium, the signals recorded for NOESY NMR referred to all the –CH_3_ groups which eventually exchange signals between the range of 0.2–8 ppm. Approximately, NOESY cross-peaks were assigned to PC-37 as well as tacrine. Most of the NOESY were intraresidue with the aromatic ring of AIL with the anti-Alzheimer's drugs. The interaction between the anti-Alzheimer's drugs and AIL *i.e.*, PC-37 + 4-PyC8/tacrine + 4-PyC8 was determined in aqueous solution and their respective ^1^H-NMR, COSY and NOESY spectra are depicted in Fig. S3,†4 and 5.

The intermolecular nuclear Overhauser effect correlation is shown in the NOESY contour between the hydrogens of the PC-37 + 4-PyC8/tacrine + 4-PyC8 residue and the hydrogens of the anti-Alzheimer's drugs (Fig. 5). It may be observed at room temperature that the NOESY resolution increases along with several cross-peaks, possibly due to decreasing tumbling rates. The correlations between the anti-Alzheimer's drugs + AIL protons reveal the complexation involved through the cross-peaks. A few aromatic rings, alkyl chains and pyridine rings in the anti-Alzheimer's drugs range were observed as NOESY cross-peaks, supporting the formation of a pre-dominant structure. Furthermore, the relative intensities of the first cross-peaks related to the anti-Alzheimer's drugs were higher in comparison to the AIL cross-peaks, which may be due to the eclipsed structure. Weak-to medium nuclear Overhauser effect cross-peaks between the hydrogens of AIL (*δ* 4.48) and both the anti-Alzheimer's drugs (*δ* 0.7–2.5 and *d* 3.0–5.0) can also be observed in the NOESY contour maps.

### 4.8 Non-binding interactions involved in complexation

Issues related to drug solubilization and structural stability are still the root cause of issues and provide the reasons to develop the present available processes. However, a lot of research has been undertaken which focuses on the study of the ILs' ability to *in vitro* solubilize drugs for stable complexation. It has been discussed earlier that functionalization of ILs results in elevation of their thermal stability, self-aggregation properties are enhanced due to the elongated alkyl chain. It has been observed that the interactions of drugs with AIL are dominated by coulombic and non-bonding forces with anions, mostly from van der Waals interactions with cations. The interaction involving ILs derives its strength from the size of the ions as well as the extent of the surface charge on the ion. The interaction between the ILs' ion moieties (either hydrophilic or hydrophobic) with the drug entities is more prominent. The interactions between ILs and drug entities help to modify the solubilization, therefore, electrostatic interactions between the drugs' hydrophilic groups are believed to be more responsible for it. This occurs due to the dielectric shielding between drugs provided by the hydrogen-bond networks. Moreover, if the hydrogen bonding between water molecules is weaker, than they are more liable to disruption by temperature and solute effects.

## 5. Summary and future outlook

Functionalized ILs by virtue of their remarkable biological properties and binding affinity towards drugs can now be successfully used as a potential tool for drug delivery systems. In addition to the inhibition efficacy of ILs towards amyloid fibrils, the interaction of synthesized anti-Alzheimer's drugs with AIL has evoked interest and a detailed investigation of the interaction process has been carried out. The present article has aimed to elucidate the drug-AIL complexation mainly by employing UV-vis, steady-state fluorescence, FTIR, ^1^H NMR, COSY and NOESY spectroscopy. The UV-vis spectroscopy results validate the formation of the anti-Alzheimer's drug-AIL complex. The negative value of free energy, Δ*G*, indicates that both the anti-Alzheimer's drugs (tacrine and PC-37) spontaneously bind with AIL (4-PyC8). Notable changes observed in the original spectrum of the AIL and anti-Alzheimer's drugs *via* multispectroscopic techniques corroborate their complexation. Spectroscopic studies reveal stronger drug–IL interactions in the case of PC-37 compared to tacrine. Absorption bands observed in the FTIR spectra of the pure drug and the mixture of AIL and drugs suggested the formation of a new complex. The difference in chemical shifts provided by ^1^H NMR data manifests significant interactions. COSY studies showed complexation in a 1 : 1 ratio and the NOESY contour shows the intermolecular NOE correlation between the hydrogens of PC-37 + 4-PyC8/tacrine + 4-PyC8. The present study frames the first picture of the complexation of these novel ILs with anti-Alzheimer's drugs. The present investigation is expected to generate exciting biochemical results with the possibility of drug permeation and pharmaceutical implementation of drug–IL complexes in clinical trials to benefit upcoming bio-medical fields. Additionally, the better understanding of the factors provided by this investigation is expected to be helpful for AD treatment. These complexes could be tuned with doped quantum dots and their feasibility and biological potential will be tested as part of our future endeavours.

## Funding

SS sincerely acknowledges the financial assistance of the Department of Science and Technology (DST), New Delhi, India for the research grant under the scheme DST INSPIRE Fellowship (Vide Order No. DST/INSPIRE/03/2016/000619). She also duly acknowledges the DST-FIST [No. SR/FST/CSI-259/2014 (C)] and UGC-SAP [No. F-540/7/DRS-II/2016 (SAP-I)] for financial support.

JK acknowledges support from a project from the Czech Science Foundation (no. 20-29633J), by MH CZ – DRO (University Hospital Hradec Kralove, No. 00179906), from Faculty of Military Health Sciences (long-term development plan).

NS and KK express their deep gratitude to the University of Hradec Kralove (Faculty of Science, VT2019-2021) and Excellence project UHK for their financial support.

## Conflicts of interest

The authors declare that they have no potential conflict of interest.

## Supplementary Material

RA-010-D0RA06323A-s001
