# Supplementary material for: Multi-spectroscopic monitoring of molecular interactions between an amino acid-functionalized ionic liquid and potential anti-Alzheimer's drugs
Source: RSC Adv. 2020 Oct 23;10(64):38873–83. doi: 10.1039/d0ra06323a (PMC9057349; doi:10.1039/d0ra06323a)
Supplement: RA-010-D0RA06323A-s001 [file RA-010-D0RA06323A-s001.pdf]

Electronic Supplementary Material (ESI) for RSC Advances.
This journal is © The Royal Society of Chemistry 2020

Supporting Information

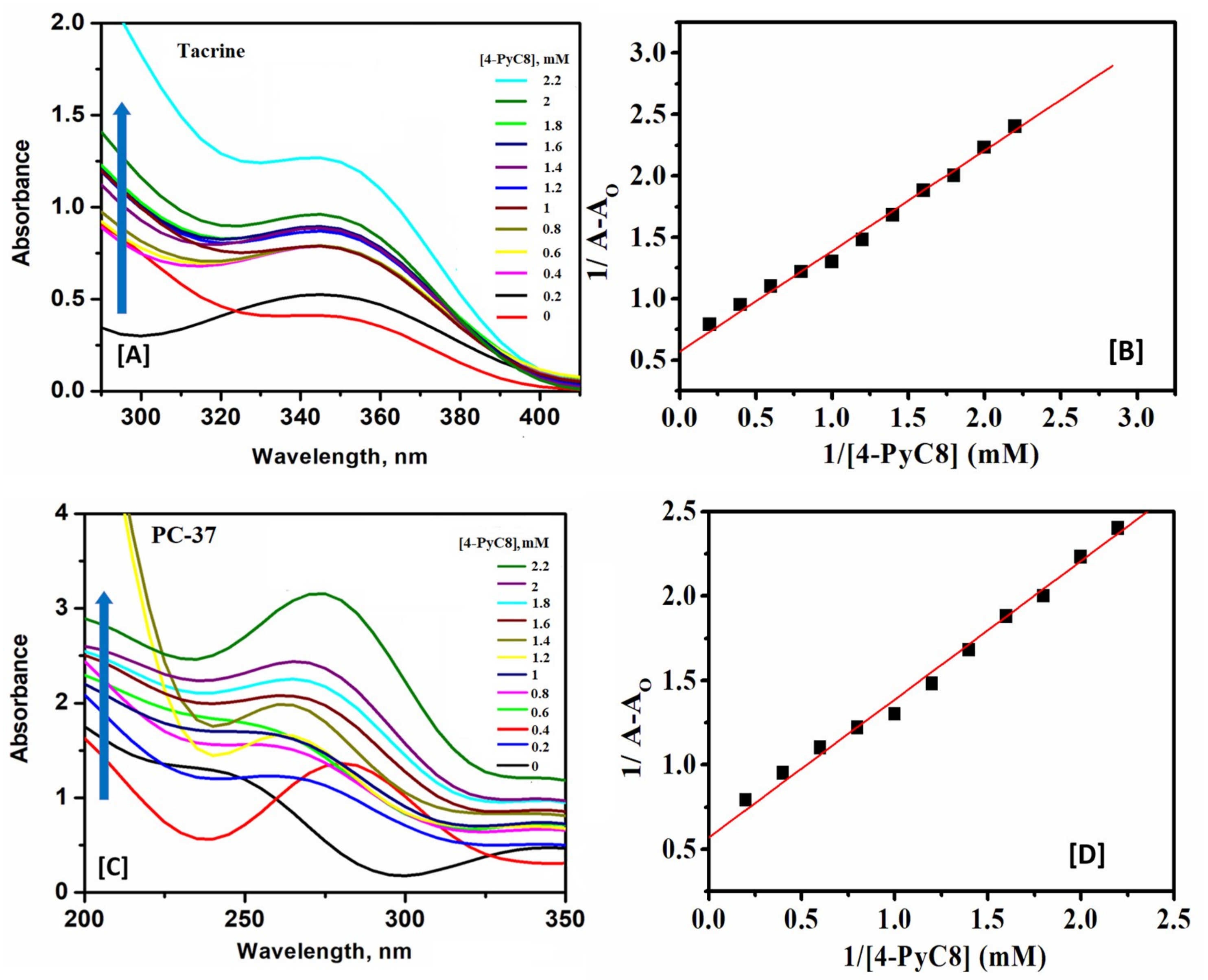

Fig. S1 Absorbance spectra (A and C) and Benesi–Hildebrand plots (B and D) for the interaction of anti-Alzheimer's drugs (1.0 mM) (A-B) tacrine and (C-D) PC-37 in the presence of increasing concentration of 4-PyC8 at physiological conditions.

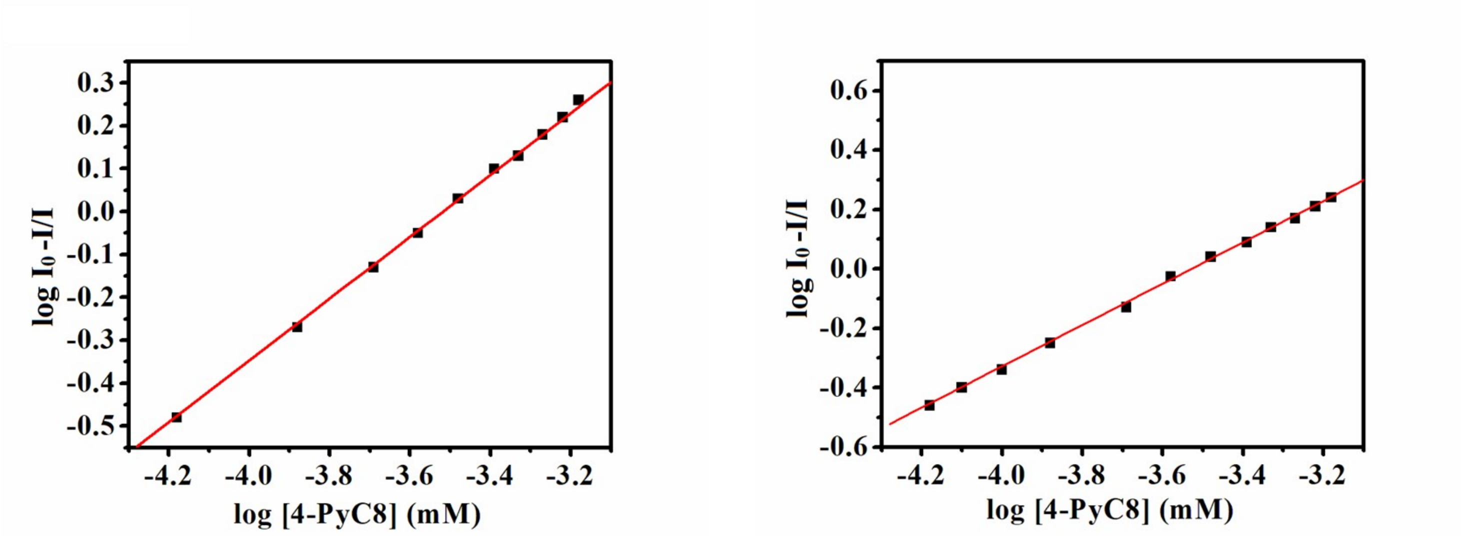

(A) **(B)**

Fig S2. Plots of log [(Io-I)/I] against log [4-PyC8] respectively for (A) tacrine and (B) PC-37 at different concentrations of 4-PyC8 (0.1 mM) at pH 7.4 and 310 K

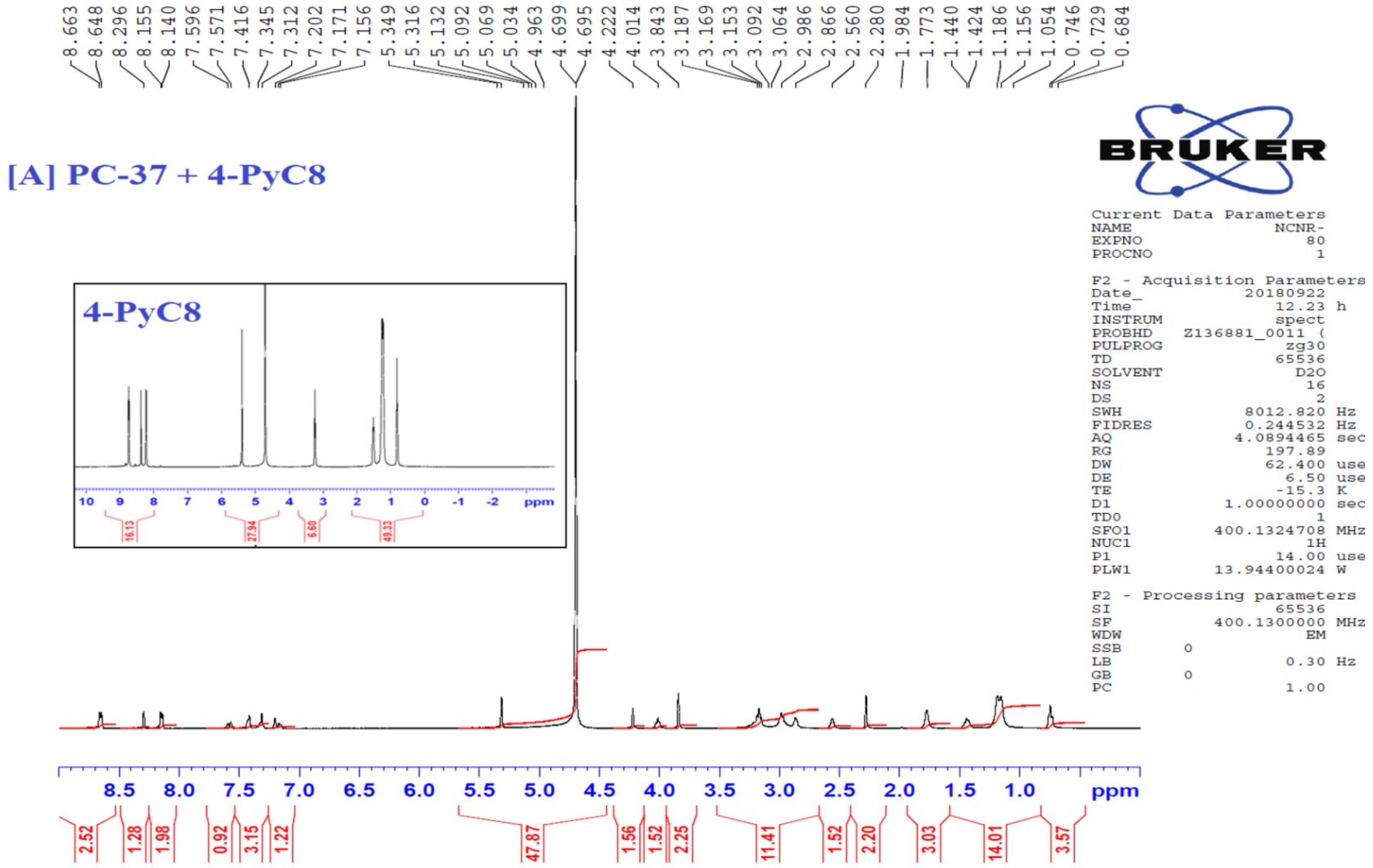

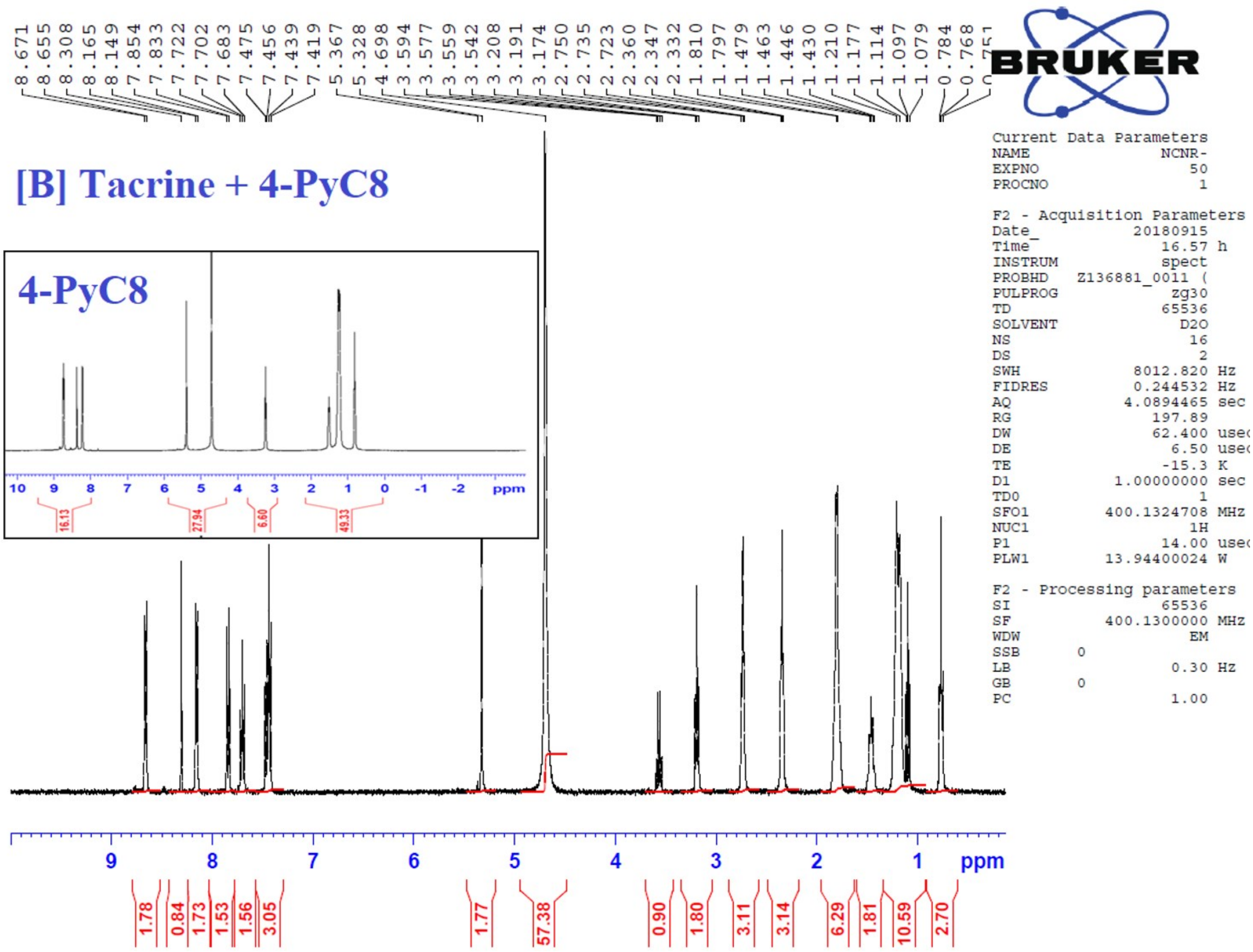

Fig. S3 ^{1}H NMR spectra of complexes of [A] PC-37+4-PyC8 [B] Tacrine+4-PyC8.
